# Quantitative and Qualitative Usage Data of an Internet-Based Asthma Monitoring Tool

**DOI:** 10.2196/jmir.6.3.e23

**Published:** 2004-09-03

**Authors:** Jacob Anhøj, Lene Nielsen

**Affiliations:** ^1^AstraZeneca A/SBusiness CommunicationAlbertslundDenmark; ^2^Copenhagen Business SchoolDepartment of InformaticsFrederiksbergDenmark

**Keywords:** Internet, asthma, self care, physician-patient relations, computer-assisted decision making, human-computer interaction

## Abstract

**Background:**

In May 2000, AstraZeneca launched a Web service for asthma patients and health-care providers called LinkMedica, which includes an asthma diary for monitoring and self-management. In the diary, the patient enters his or her peak flow, number of doses of rescue medication, and if there have been any asthma symptoms during the previous 24 hours. The patient receives an immediate response from LinkMedica, telling him or her if the asthma is under control and what to do if not, eg, increase the dose of inhaled steroid. Health-care providers have access to the patient diary.

**Objectives:**

The primary objective of the study was to describe patients' and health-care providers' use of LinkMedica. Secondary objectives were to evaluate their perception of the system and how the users' interaction with the system is influenced by their everyday lives.

**Methods:**

Site statistics regarding number of registered users and diary usage were analyzed. An online survey among users (85 respondents), a mailed questionnaire to health-care providers (131 respondents; response rate 26.8%), as well as in-depth interviews with 10 patients and 5 general practitioners, elicited further quantitative and qualitative data on users' perceptions.

**Results:**

In February 2003, a total of 7653 users had registered. During 2002, the growth in registered users averaged 50 per month. In the same period, the number of unique diary users per month decreased from 307 to 138. Patients usually stopped using the diary after a short time; the doctors were reluctant to introduce the diary to patients because of time constraints. Several user subtypes were identified among patients and their relatives.

**Conclusion:**

The self-selected survey responses and in-depth interviews indicated that LinkMedica is generally considered a trustworthy and reliable site by both patients and doctors. However, there was a contrast between users' positive perception of LinkMedica and their unwillingness to use the site for more than short periods. The primary reason for this was that LinkMedica did not fit into their everyday lives because of technical and psychological aspects. A number of recommendations to improve LinkMedica are suggested.

## Introduction

The cornerstone of modern asthma care is self-management, allowing the patient to monitor his or her disease severity continuously and to adjust the dose of inhaled corticosteroid based on symptoms, lung function, and use of rescue medication [1]. A recent Cochrane Review concluded that self-management might improve asthma outcomes significantly [2]. Several strategies have been developed, including patient education and written actions plans.

With the appearance of the World Wide Web, new opportunities for communication and interaction between patients and health-care providers have emerged. The Internet has been suggested as a tool for monitoring and for self-management of a number of chronic diseases, eg, diabetes, hypertension, and asthma, and a small number of studies has been reported [3-8].

LinkMedica DK was launched in May 2000 as a Web service for asthma patients and health- care providers. The service enables asthma patients to monitor their condition using an electronic asthma diary, and allows health-care providers to access their patients' diary data. LinkMedica was sponsored and designed by AstraZeneca Denmark in cooperation with the Danish Asthma and Allergy Association and an independent advisory board of asthma specialists.

To our knowledge, LinkMedica was one of the first publicly available services taking advantage of the Internet for self-management of asthma and allowing health-care providers to access patient diary data online, thus improving and facilitating the cooperation between health-care providers and patients.

The LinkMedica Web site is currently available in Denmark [9]. Until 31 March 2004 LinkMedica was also available in the UK [10]. Besides different languages, the main difference between the two sites was different algorithms controlling feedback messages to patientsin the diary. This reflects the fact that LinkMedica is prepared for localized set-ups in different countries with different clinical guidelines and treatment practices.

**Figure 1:**
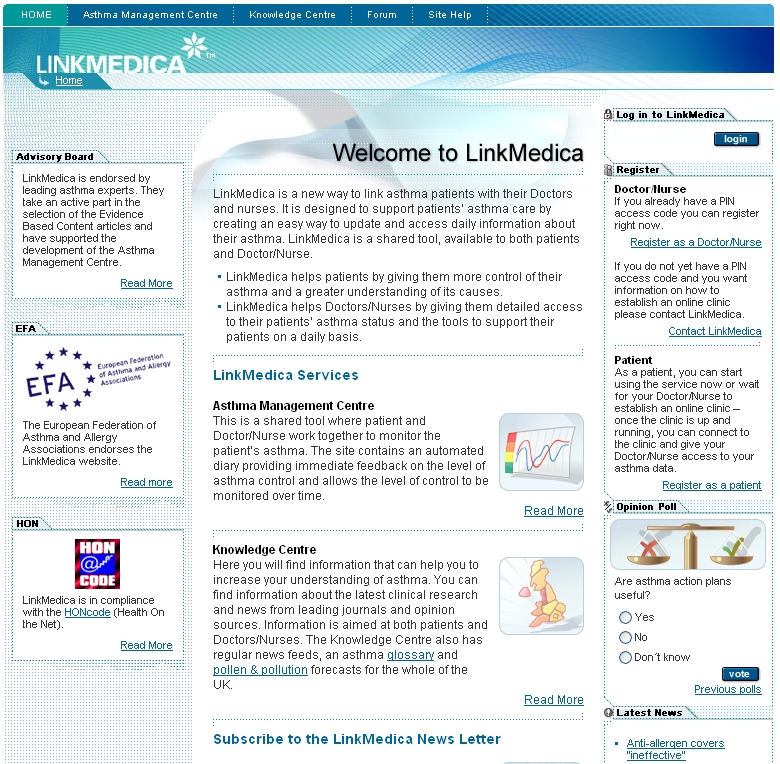
Screenshot from linkmedica.co.uk

Below, LinkMedica is briefly described. Readers are encouraged to visit www.linkmedica.dk (Danish) for personal study. Fig. 1 shows a screenshot from linkmedica.co.uk

### System Description

LinkMedica has three main sections: Asthma Management Centre (AMC), Knowledge Centre (KC), and Forum. KC and Forum are immediately available to everyone, whereas AMC requires the user to register and create a user name and a password. Patients are able to create their own accounts online, while health-care providers are required to contact AstraZeneca to get registered. This is to confirm the identity of doctors and nurses available to patients on LinkMedica. When a patient has registered, he or she may select one or more doctors or nurses from the list of available health-care providers. This grants the health-care providers access to the patient's asthma diary if he or she accepts the patient. This procedure ensures that both parties have accepted their collaboration via LinkMedica. After the doctor or nurse has accepted or rejected a patient, the patient receives a notification about this at next login.

#### LinkMedica Asthma Management Centre

AMC contains the asthma diary. The intention is that the patients log on every day and enter their asthma values: morning peak flow, number of doses of rescue medication, and whether they have had asthma symptoms at night. After submitting diary values, the patient receives an immediate response saying whether or not his or her asthma is under control and, if it is not, providing detailed instructions on what to do. For example, the user may be instructed to double the dose of inhaled corticosteroids for a period of two weeks if he or she has reported asthma symptoms on two consecutive nights. AMC also has graphics that show trends in peak flow and symptoms coupled with environmental factors such as pollen counts and air pollution.

When a health-care provider logs on to LinkMedica, he or she is shown the list of patients who have permitted him or her to access their diary data. By clicking on a patient's name, the health-care provider can see that patient's diary data and graphs.

#### LinkMedica Knowledge Centre

In KC, users can find a large number (>100) of articles and news about asthma and allergies. The article section contains summaries of evidence-based scientific papers from peer-reviewed journals. These summaries are presented in two formats: 1) "In summary," a user-friendly summary of scientific papers, written in consumer language, and 2) "In detail," a fuller version of the scientific paper and link to the published paper abstract.

The following process is used to select papers: A project coordinator appointed by a subcontractor (Foresight Links Corporation) oversees the selection process. The coordinator, who has professional expertise in evidence-based decision-making, is the main liaison between Foresight Links Corporation, the LinkMedica team and the advisory board.

First, the coordinator conducts a search of the databases of "distilled" evidence using "asthma" as the only keyword, and produces a list of the citations yielded by the search. 

Databases used include The Cochrane Database of Systmatic Reviews, Best Evidence, Database of Abstracts or Reviews of Evidence (DARE), Clinical Evidence, and Bandolier.

These databases have been built up over the last 10 years by internationally recognized initiatives (including professional and governmental organizations such as the American College of Physicians, the British Medical Association, and the Cochrane Collaboration), engaged in the collection, appraisal, and synthesis of the best available evidence from clinical research.

As a second step, this list of citations is sent to the advisory board members, together with the list of topics identified as relevant to patients and health professionals. The members of the advisory board are asked to select articles covering as many of the topics on this list as possible. When there are no articles on the list to address topics, members of the advisory board are invited to identify "classic" or recent articles published in peer-reviewed journals, based on their own content knowledge and expertise. These articles are retrieved from the Cochrane Controlled Trials Database and PubMed, and are included only if they meet the selection criteria used by ACP Journal Club. The full article selection process was done before launch of LinkMedica UK in summer 2001. Every four or five years, articles will be reviewed again by the advisory board members who are responsible for ensuring the content is up-to-date.

In addition, news from Danish and international media of interest to asthma and allergy patients is being added on a regular basis from Observer Denmark [11].

#### LinkMedica Forum

In the Forum section, users may participate in unmoderated discussion groups and ask questions, which are dealt with by experts. The experts are advisers from the Danish Asthma and Allergy Association, a specialist in environmental medicine, a pediatrician, a dermatologist, and a general practitioner [12]. AstraZeneca and the Danish Asthma and Allergy Association selected the experts.

#### Objectives of This Study

The primary objective of this study was to describe patients' and health-care providers' use of LinkMedica. Secondary objectives were to evaluate users' perception of LinkMedica and how their everyday lives interact with the system.

## Methods

A total of four user studies (two surveys, two interview rounds) were launched in summer/fall 2002. Site statistics were evaluated in February 2003.

### Site Statistics

Site statistics are available to the site administrator online from LinkMedica's back end based on Web site log files. Total number of registered users at the end of each month and number of unique diary users each month (number of users entering diary values at least once per month) were extracted and plotted against time for visual inspection.

### Surveys

Two user surveys were carried out. The first was an online pop-up survey targeted at all users visiting the site from April 29 to May 30, 2002. When visiting LinkMedica during this period, the user was presented with a pop-up window asking the user if he or she was willing to participate in an online survey. Except for the introductory text, a yes and a no button were the only elements on the pop-up form. If the user pressed the no button, the window was closed. If the user pressed the yes button, he or she was redirected to the survey form. Regardless of the button pressed, a cookie was set on the user's hard disk to prevent the pop-up window appearing on subsequent visit. If the pop-up window was closed by other means-eg, by clicking the cross in the upper right corner-no cookie was set. No attempts were made to prevent users from submitting more than one survey form by filling in the form on different computers or by deleting the cookie from their own hard disk. We assumed, however, that the risk of a significant number of users doing this was negligible.

The response rate was not monitored, but the number of submissions was compared with the number of unique visitors (by IP-address) in May 2002.

The survey questionnaire contained 17 questions. In this article we present the results of 7 selected questions: "Your age?"; "Your gender?" (male, female); "What is your background?" (patient, relative, health care professional); "What is your primary reason for visiting LinkMedica?" (seeking information, seeking advice, asthma diary); "How often do you visit LinkMedica at present?" (daily, weekly, monthly, less than monthly); "How often do you intend to visit LinkMedica in the future?" (daily, weekly, monthly, less than monthly); "How do you rate the quality of LinkMedica?" (very good, good, poor, don't know). The last question was asked for each subsection of LinkMedica (diary, knowledge centre, forum).

Complete results from the full questionnaire are available in an internal AstraZeneca report, which is available free (in Danish) to anyone interested.

Males and females were compared with respect to age distribution, reason for visiting LinkMedica (information, advice, diary), and user's background (patient, relative, health-care provider).

The second survey, a mailed questionnaire, was sent to all health-care providers that- according to AstraZeneca's customer database-had received a user name and password for LinkMedica. The questionnaire was in two sections. The first section of 4 questions was intended for all respondents. The second section of 15 questions was intended for those who, in their own opinion, had ample experience in using LinkMedica. Only the results from the first section are presented in this article. The full report (in Danish) is available free to anyone interested. The questions from the first section were: "Your profession?" (physician, nurse, secretary, other); "Have you heard of LinkMedica?" (yes; no); "Do you think that there is a need for Internet tools like LinkMedica in medical practice?" (yes, no); "Do you ever use LinkMedica in collaboration with your patients?" (Yes-frequently, Yes-sometimes, I have looked at it-but did not find it useful, No, No-but I would like to try).

The questionnaire results allowed us to select persons representing different types of LinkMedica users for interviews; respondents of both surveys were asked to provide name, address, and phone number if they were interested in being interviewed.

### Semi-structured Interviews

To seek to understand the social world as it is for those people whose social world it is, is possible only if one practices the art of listening to them in their own terms and attends to the social world they construct for themselves. (Zaner [13])

In-depth qualitative interviews were conducted with users in order to get an understanding of the patients' approach to their illness and their use of the Internet and the doctors' approach to the use of Web-based monitoring systems.

A total of 15 users were selected from respondents who were willing to be interviewed for semi-structured interviews: 8 patients, 2 mothers of children with asthma, and 5 GPs. They were selected to represent: 1) male and female users, 2) users of AMC and of KC and Forum, 3) frequent visitors and occasional visitors, and 4) health-care providers, patients, and relatives.

The interviews were designed to address three issues:

- Who are the users?
- How do the users use LinkMedica?
- How do the users' everyday lives interact with LinkMedica?

The perspective of the qualitative method is to understand the world as inter-subjective-to understand the world from the point of view of those who live in the world. The purpose of this interpretative approach is to understand social phenomena-to understand the lived experience and the complex world this experience takes place in [14,15].

All interviews were conducted as semi-structured qualitative interviews according to Kvale's criteria for conducting and analyzing qualitative interviews [16]. They were taped and transcribed. Each interview was broken down into thematic units and these were compared across interviews. As the qualitative method provides insight into the inter-subjective world, it is not possible to quantify the data: they are interpreted as themes from the lived experience of those interviewed.

The starting point of the interviews was the patients' relation to their disease, and both doctors' and patients' strategies for information seeking and use of the Internet. Themes that appeared during the first interviews were pursued in later interviews.

## Results

### Trends in Number of Registered Users and Diary Users

In February 2003, a total of 7653 diary users were registered on LinkMedica. The number of unique diary users at that time was 138 per month.

**Figure 2:**
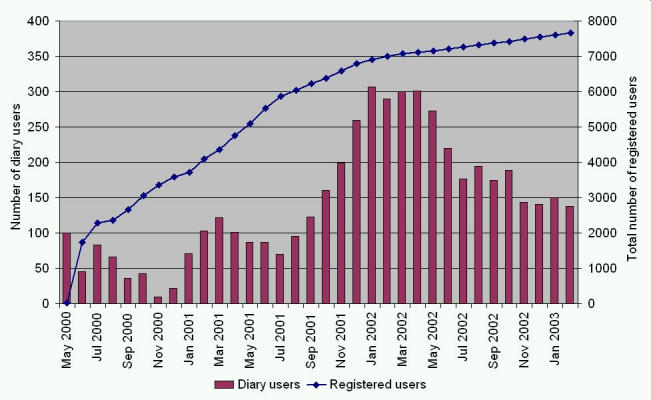
Number of unique users that have entered their diary per month (bars) and total number of registered users (line)

Figure 2 shows that the growth rate of user numbers falls into three phases: In the first 3 months after launch, more than 2000 new users registered. During the next year and a half approximately 4000 users registered, and during the last year there was a growth of approximately 50 new users per month.

The trend in unique diary users is more uneven. After a peak of 100 diary users in May 2000, the number decreased until November 2000, when only 9 users kept an online diary. During the next year the number of diary users increased (with a decrease during holiday seasons) to a maximum of 307 in January 2002. This increase occurred at the same time as AstraZeneca started marketing LinkMedica. After this, the number of unique diary users was steady for a period of about 4 months. However, in February 2003, the diary user number had declined to 138 per month.

### User Survey

Between April 29 and May 30, 2002, 85 users responded to the online pop-up survey. Compared to a total of 3689 unique visitors in May 2002, this gives an estimated response rate of 2.3%. Of these 59 (69%) were patients, 12 (14%) were mothers of children with asthma and 8 (9%) were health-care providers (see Table 1). Inter-quartile age range was 29 to 43 years.

**Figure 3:**
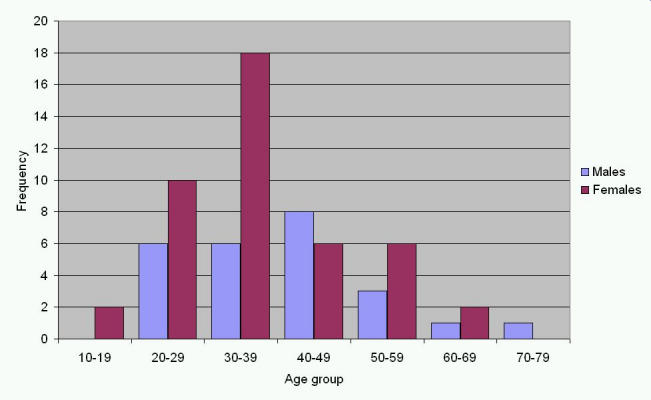
Age distribution of users

Because of a programming error in the survey, gender was not recorded for 13 users. Of those remaining, two thirds were female. Mean age was 36 years for females and 41 years for males. This difference was not statistically significant (p=0.12). The age distribution for males and females respectively is shown in Figure 3.

User background and reason for visiting LinkMedica differed somewhat between males and females. Most females were seeking information and advice (49%), while the majority of males gave the asthma diary as primary reason (56%) (Table 2). Only 1 of 7 health-care providers was female. This difference between male and female users with respect to their background (patient, relative, health-care provider) and their reason for visiting LinkMedica (information, advice, diary) was statistically significant (p<0.05, chi square test).

Seventy-two percent reported that they visited LinkMedica at least once a month, and 92% reported that they expected to visit LinkMedica at least monthly in the future.

When asked how they perceived the quality of LinkMedica, the majority of users answered that the quality was good or very good. However, for each main section a rather large percentage of users answered that they did not know (Table 3).

**Table 1:** Demographics of respondents to pop-up online survey

Background	All	Males**	Females**
Patient	59 (69%)	17 (68%)	32 (68%)
Relative	12 (14%)	0 (0%)	11 (23%)
Health care professional	8 (9%)	6 (24%)	1 (2%)
Other	6 (7%)	2 (8%)	3 (6%)
Total	85 (99%)*	25 (100%)	47 (99%)

^*^ Because of a programming error in the survey, gender could not be accounted for in 13 users

^**^ p = 0.04, chi square test

**Table 2:** Response to the online pop-up survey question "What is your primary reason for visiting LinkMedica?"

Reason	All	Males*	Females*
Information (Knowledge Centre)	23 (27%)	4 (16%)	18 (38%)
Advice (Forum)	6 (7%)	0 (0%)	5 (11%)
Asthma diary (Management Centre)	39 (46%)	14 (56%)	18 (38%)
Other	17 (20%)	7 (28%)	6 (13%)
**Total**	85 (100%)	25 (100%)	47 (100%)

^*^ Because of a programming error in the survey, gender could not be accounted for in 13 users

**Table 3:** Users' responses to the online pop-up survey question: "How do you rate the quality of Asthma Management Centre, Knowledge Centre, and Forum respectively?"

Assessed quality	Asthma Management Centre	Knowledge Centre	Forum
Very good	27 (33.7%)	15 (18.7%)	8 (10.3%)
Good	28 (35%)	33 (41.2%)	24 (30.8%)
Poor	2 (2.5%)	2 (2.5%)	2 (2.6%)
Don't know	23 (28.7%)	30 (37.5%)	44 (56.4%)

### Health Care Providers Survey

Out of 489 questionnaires mailed to health-care providers that-according to AstraZeneca's customer database-had been given user name and password for LinkMedica, 131 were returned (response rate 26.8%). Among the respondents there were 127 (97%) physicians and 4 (3%) nurses. Fifty-one (39%) used LinkMedica intermittently or frequently for their asthma patients. Questionnaire results are summarized in Table 4 and Table 5.

**Table 4:** Health-care providers' answers to two questions from the mailed questionnaire

**Question**	**Yes**	**No**	**Total**
Have you heard of LinkMedica?	113 (86%)	18 (14%)	131 (100%)
Do you think that there is a need for Internet tools like LinkMedica in medical practice?	96 (73%)	35 (27%)	131 (100%)

**Table 5:** Health-care providers' answers to the question: "Do you ever use LinkMedica in collaboration with your patients?" (from mailed questionnaire)

**Answer**	**Frequency**
Frequently	4 (3%)
Sometimes	47 (36%)
I have looked at it-but did not find it useful	4 (3%)
No	46 (35%)
No-but I would like to try	29 (22%)
Total	130 (99%)

### Interviews

A total of 15 one-to-one in-depth interviews, each lasting approximately 60 minutes, were conducted.

#### Who Are the Users?

Five thematic types of users were identified among the interviewees. Characteristics are summarized and compared in Table 6.

**Table 6:** Interviewees

User	Demography
Patient 1	Male, 28, severe asthma, uses the diary. Has posed questions to expert.
Patient 2	Female, 37, asthma, diary user, mother of two, one has asthma.
Patient 3	Female 33, asthma, mother of a child with asthma, does not use the site.
Patient 4	Female 53, severe asthma and allergy, diary user.
Patient 5	Female 43, asthma and allergy, has used the site for information.
Patient 6	Male, 40, asthma, diary user.
Patient 7	Female, 35, asthma, diary user, pregnant with first child.
Patient 8	Female, 38, daughter with allergy, has posed questions to expert.
Patient 9	Female, 38, son with severe asthma, uses the whole site.
Patient 10	Male 48, asthma, diary user.
GP 1	Female 40, been a GP for 3 years, uses the diary.
GP 2	Male 53, been a GP for 18 years, uses the diary.
GP 3	Male 52, been a GP for 26 years, uses the diary.
GP 4	Male 52, been a GP for 20 years, does not use the diary.
GP 5	Male 58, been a GP for 20 years, working part time, uses the diary.

#### Patients

We identified a thematic difference amongst the patients. We labeled the two distinctly different types of patients as *controllers* and *neglecters*.

The **controllers** wish to gain control of their disease. They establish daily routines to control and monitor the disease so they do not have to worry about it. Their homes are designed to prevent asthma attacks. They use AMC to monitor their condition.

The following is an excerpt from a patient interview. Interviewees are listed and characterized in Table 7.

Interviewer: "Almost everybody in the family suffers from asthma. Does it influence your everyday life?"

Patient 2: "No, not really. It influences us in that way-as you can see-that we do not have carpets, just the bare floor. And our son cannot have the pets that he would like to have. And we have installed a ventilating system in order to try and reduce the humidity. So in that way it has influence on our surroundings. We have chosen to hire a cleaner to clean the house because we realize that we cannot do it properly ourselves. So it does influence our lives, but we do not think about it on a daily basis."

The **neglecters** do not want to think about their disease. By not focusing on it, they feel better. In this way they do not use mental energy on the disease, and they consider this to be good for their health. To feel secure, they just need to carry their rescue medication with them. This excerpt from an interview reflects this attitude.

Patient 4: "Then I must figure out myself what is good for me.

Interviewer: "Instead of exploring and reading?"

Patient 4: "Yes-you can get so focussed on it at times. Sometimes it is better to pretend nothing is wrong. It's a balance, you know."

There was no distinction between the sexes in these attitudes.

We also observed that different user types might very well be expressed within the same person at different times and that most users possess traces of both the controller and the neglecter types. Thus, a person could say that he or she did not pay attention to the disease and at the same time talk about refurbishing the entire house or about being highly aware of things that might provoke an attack.

#### Mothers

The two mothers of children with asthma or allergy interviewed were different in their needs and response patterns to information. One motherexpressed an urgent need for information and responded emotionally to the information. She was mainly interested in guidelines that could help her in her present situation. She was not interested in abstract knowledge such as research results or scientific information.

The other mother used all her energy to control her child's disease. She subscribed to news and was active in the Forum. She was empowered by the use of AMC and strived to control her child's disease. She did not think this behavior influenced her family life. The following excerpt is an example of this.

Interviewer:" I can see you spend a lot of time looking for information. How does that influence your everyday life?"

Patient 9: "It does not-in any negative way. I think it is good that I don't have to spend all day reading newspapers. Now I can use the Internet if there is anything I need to know."

These two mothers were comparable to the neglecter and the controller types. They will be referred to as the **emotional** and the **professional** mother respectively.

#### General Practitioners

The typical GP user is a male around fifty years old who works at a GP clinic with a small number of other doctors. Two thematic subgroups were identified: the *user*, who had experience in monitoring asthma patients with LinkMedica and the *interested*, who was considering using LinkMedica in the future.

The **user** was introduced to AMC through participation in a clinical trial. He finds the system of great value for the patients, but he doesn't use it himself now the trial has ended. He finds that AMC has too many functions-more than he needs. He finds the system to be complex with a complicated login procedure.

The **interested** has no experience in using LinkMedica but has heard about it. He believes that both he and his patients might benefit from using the system.

The typical GP user is not a confident user of either the Internet or a PC. The GP knows his own electronic patient record system, but he doesn't use the PC for anything else. He is connected to the Internet through an integrated services digital network (ISDN) connection. This creates obstacles for a smooth login procedure and prevents him from being online all the time.

**Table 7:** Thematic user segmentation

User segmentation	Patient: The disease controller	Patient: The disease neglecter	Relative: the emotional mother	Relative: the professional mother	The GP
Cause & action	Causal (it is possible to find the cause for an attack/rash)	Deterministic (the causes can be found but they are not important to find)	Causal (it is possible to find the cause for an attack/rash)	Causal (it is possible to find the cause for an attack/rash)	No focus on the causal relationship
Relation to disease	Instrumental	Ad hoc information seeking according to need	Instrumental	Ad hoc information seeking according to need	Patient must learn to accept their asthma
Knowledge	Disseminated information.Active search for information	Experienced information.Information is sought when acute needs appear.	Disseminated information.Active search for information	Experienced information.Information is sought when acute needs appear.	Courses arranged by the Danish Medical Association or the medical industry
Information type	Research articles, news, etc.	Funny information, clarifying information.	Instructive information	Research articles, news, etc.	Medical journals, easy read articles
Relation between GP and patient	The GP is perceived as ignorant on subjects concerning asthma and allergy	The GP is perceived as ignorant on subjects concerning asthma and allergy	The GP is perceived as ignorant on subjects concerning asthma and allergy	The GP is perceived as ignorant on subjects concerning asthma and allergy	The GP is a consultant for the patient

#### How Do Users Use LinkMedica?

From the interviews we found that the user's perspective plays an important role in how the system is perceived and used.

In the **outside-in** perspective, the users have a problem that arises in the outside world and expect to find answers in LinkMedica. These users have an acute need for information and browse the site to fulfill this need. They ask questions, and they expect a quick reply. It is mainly women who have the outside-in perspective.

In the **inside-out** perspective, the users focus on the use of the diary. They do not read or look for articles and news. But once in a while, an interesting headline may catch their attention. They expect the system to operate as smoothly and as quickly as possible. They have high expectations to the usability of the system. Most of the users who used the diary participated in another research project. It is more often males that have the inside-out perspective. This also applies to GPs.

#### How Does the User's Everyday Life Interact With LinkMedica?

Most patients interviewed found it easy to use LinkMedica and to enter diary data. In spite of this, none of them used the diary as intended, ie, entering diary values immediately after measuring morning peak flow. All patients wrote the values on a piece of paper and entered the values in LinkMedica whenever it was convenient. Some had access to the Internet at work while others used their home computer in the evenings or during the weekends.

In general, we found that the users were satisfied with LinkMedica; some expressed a will to continue using the site to monitor their asthma over time and to identify asthma triggers. But even though they were motivated, most of the users believed that they would not continue using the site. This excerpt shows a typical behavior:

Patient 2: "I do not enter diary values every day. I do it in batches. On the other hand, you should enter values every day in order to benefit from the system. I really want to, but I never get it done."

As a system, AMC is seen as a reliable tool. However, when the patient receives an unexpected message, one that contradicts his or her previous experience, the patient reacts with disbelief. The patients that had experienced a red alert instructing them to increase the dose of inhaled steroid did not understand why this was important, and none of them took the prescribed action.

In general, the patients were reluctant to use medication on a regular basis. It was difficult for them to accept the fact that a daily dose of medicine is better than using medicine only when experiencing symptoms, as this excerpt shows:

Patient 6: "I thought it was a high dose. I did follow the instructions on the Internet-but not the dose."

Interviewer: "Why not?"

Patient 6: "I might have had a low peak flow for a couple of days. But it kept instructing me to increase the dose, and I did not think it was necessary.

Interviewer: How much would be reasonable?"

Patient 6: It should have said a little bit more.... I do not know the side effects [talking to the microphone]-do I?

When asked to consider the ideal patient for the system, the GPs generally described a young man who does sports and has an interest in computers, as expressed here:

GP 4: "I imagine a young man around 20 who is troubled by his asthma and knows about computers. So I don't have to explain everything to him. I think it is complicated and I would rather not explain it."

The GPs said that their relations with the patients are currently in transition. From being considered experts, doctors are now more like consultants who identify problems and cures in collaboration with the patients**.** The GPs find that this is a positive development. But resources are limited, and the consultations still have to fit the 10-minute slots GPs can make available for appointments. This puts a strain on the GPs. Furthermore, as most GPs themselves are not confident PC users, they find it very difficult to instruct the patients in the system. The consultant role requires confidence as well as technical insight, and most GPs do not feel they have this insight.

From the GP's point of view, the patients benefit from using LinkMedica. The system helps patients understand their disease, improves compliance, and reduces symptoms. Furthermore, LinkMedica stresses the patient's own responsibility for his or her disease, as this GP says:

GP 5: "It is really motivating. The first of my patients who used the system came back to me and said: I'm so happy I tried this. I went hill-walking in Norway, and you know what? I went all the way to the top and back down again. I haven't been able to do that for many years."

None of the GPs reported that the patients had difficulties in using the system. The GPs themselves, however, found that using LinkMedica was difficult. The login procedure, especially, was perceived as an obstacle. A GP expressed it like this:

"It is an obstacle. I think it is important that the computer logs you in automatically by remembering your login information. All that about changing your password: Forget it! People don't do it."

From the GP's perspective, AMC is a useful tool, especially for things that computer systems do well: record keeping and performing calculations. This is the major advantage of the system. The disadvantage is that it takes time to log in and to instruct the patients. Also, the GPs found that the system has more functions than necessary.

Even though the GPs have a positive attitude towards the system, their use of it is influenced by external factors such as time and economy. As the GPs put emphasis on these factors, they do not use the system.

## Discussion

Survey and interview data indicate that users are happy with LinkMedica in general. Patients find that the asthma diary helps them manage their disease, and doctors find that the diary improves asthma control in patients using it. This observation is supported by preliminary data from a clinical trial. These data, which are currently under evaluation and have been published in abstract form**,** suggest that LinkMedica improves lung function, asthma severity score, and bronchial hyperreactivity compared to traditional treatment regimens initiated by either a GP or a pulmonologist [17].

LinkMedica as a whole is considered a reliable system that offers information of high quality about asthma and allergy. Furthermore, doctors and patients haveexpressed a need for improved tools for asthma monitoring and management. In this respect, the users consider the Internet a medium of high interest.

Interestingly, however, we also found that despite their positive attitude and readiness, both doctors and patients usually stop using LinkMedica after a short period of time. From site statistics and informal user contacts we were already aware of this problem before the project started. The project, however, has given us insight into possible reasons forthis evident paradox:

- Users are inexperienced with the Internet and computers.
- Access to the Internet is limited and cumbersome.
- Users' everyday lives interact with LinkMedica in unpredictable ways.
- Different user types have conflicting needs.
- Internet information may support but not change users' inherent attitudes.
- The benefits of using an asthma diary are not recognized immediately.

### Users Are Inexperienced With Internet and Computers

Surprisingly, we found that the users having most difficulties with LinkMedica were the doctors, whom we expected to be confident computer and Internet users. None of the patient users expressed any difficulties whatsoever using LinkMedica. The main complaint from doctors was that the login procedure is complicated and time consuming. The doctors have difficulties managing different user names and passwords for different services. Even though changing user name and password to something easy to remember is straightforward in LinkMedica, none of the doctors did this.

From training sessions with GPs, it is our personal experience that doctors find it difficult to use more than one application at a time. In general, doctors are satisfied with their electronic patient record system available from their desktop, but they do not feel confident in "windows juggling." This prevents them from having LinkMedica at hand whenever an asthma patient comes to the clinic.

In this sense, lack of practice and confidence hinders the use of LinkMedica. But surprisingly, only doctors seem to have this problem. This observation could be the result of a selection bias, leaving only computer literate patients for survey and interview. Although this may be partially true, we do not believe this fully explains why patients apparently have fewer problems than doctors accessing LinkMedica. It may be that doctors have higher demands and are more critical because of time constraints in their work. The fact that doctors on average are older, have less experience with the Internet, and seem to use the Internet less than patients for general information seeking may also play a role.

### Access to Internet Is Limited and Cumbersome

Easy access to the Internet is critical for users' experience with LinkMedica. In 2002, 76% of Danes had access to the Internet either from work or home, and 56% used the Internet at least weekly (38% daily) [18]. Thus, in Denmark, Internet availability is hardly a barrier. But speed of connection may be. In the surveys, we did not ask how the users connected to the Internet. But in the interviews, some users complained that the connection and logon time through an analog modem was an obstacle. Although our impression is that LinkMedica loads faster than many other Web sites with comparable content, booting the computer and dialing up with a modem may take several minutes. In comparison, it usually takes less than 20 seconds to fill in the diary and receive the feedback message. As broadband connections become more available and affordable in the future, we expect these problems to diminish.

### Users' Everyday Lives Interact With LinkMedica in Unpredictable Ways

In our opinion, an interesting finding from this study is how much users' everyday lives interact with LinkMedica. As an example, take the doctors' time schedules. They have about 10 minutes per patient. This, together with the fact that most doctors do not feel confident in using the Internet, or even their computers, has an enormous impact on how the doctors look at LinkMedica. The end result is that not a single one of the doctors that we interviewed used LinkMedica on a regular basis, despite their positive attitude. Furthermore, the doctors were mistaken in their views of who would use LinkMedica. According to the doctors, the typical LinkMedica user would be a young, sporty man, when in fact, the typical user is a mother around 40 years of age.

The following is another example of how everyday life influences the use of LinkMedica: Typically, the diary users measure their peak flow in the morning and write down the value. They collect the values for a week and type them in later, during the weekend. This way, feedback messages are received days after the condition that triggered them, and the whole idea of immediate dose adjustments due to changes in symptoms or peak flow is lost.

We are convinced that this problem has something to do with the Internet still being separate from the rest of people's everyday lives. The Internet is something you actively connect to, not something that is just there like the telephone or the television. In this sense, LinkMedica and other Internet-based disease management systems are ahead of their time, and we would expect these programs to gain popularity and usability as the Internet gets more integrated into our everyday lives. Currently, we are investigating other means of connecting to LinkMedica, eg, short messaging system (SMS) and general packet radio service (GPRS). These technologies have the advantage of being closely integrated into people's everyday lives and are immediately available to anyone, anywhere without the inconvenience of having to start the computer or wait for slow dial-up connections.

### Different User Types Have Conflicting Needs

A number of patient subtypes were identified: controllers, neglecters, professional mothers and emotional mothers. The doctors came in two groups: users (or more correctly, former users) and potential users, who had no prior experience with LinkMedica but who were interested in trying it. During the interviews, it became obvious that these highly different user types have very different requirements and expectations of LinkMedica.

Different parts of LinkMedica (AMC, KC, Forum) are considered important depending on the user's perspective: inside-out or outside-in. The doctor and the controller, together with the professional mother, expect the diary (AMC) to function without a hitch. News, discussion forums, and ask-the-expert sections are merely distracting elements preventing them from having fast access to the diary. If they ask for information, they want it to be as complete as possible, enabling them to decide for themselves how to act. They prefer evidence-based articles and expert opinions to news and advice from other users. The neglecter and the emotional mother, on the other hand, seek information only when they need it. They expect concise information and concrete advice to help them in their current situation. They usually avoid scientific articles and expert opinions unless they are directly applicable to their current needs. They are not interested in monitoring their disease using the diary.

Creating a Web site that seeks to satisfy such conflicting needs and user perspectives may not be a good idea. For future Web projects like LinkMedica, we suggest that the target users be defined clearly from the very beginning. For existing Web sites of complex nature like LinkMedica, it may be worthwhile considering a split into several more focused sites, which may, of course, be interlinked.

### Internet Information May Support but Not Change Users' Inherent Attitudes

It is an interesting observation that diary users (controllers) were the ones least likely to follow the advice in the feedback messages. As mentioned previously, not a single interviewee who received an alert message instructing him or her to increase the dose of steroid followed this advice. This observation calls into question the whole idea of the diary design as it is today. If the users most likely to use the diary are also the users least likely to follow specific advice from feedback messages, it might be worthwhile reconsidering the format and the content of the feedback messages.

The idea of having an electronic asthma diary with an "intelligent" feedback system was to support and educate asthma patients in self-management. In this respect, the diary may serve as a daily consultation with a virtual asthma expert. This study taught us, however, that an important difference between virtual and real experts is that patients do not readily accept advice from a virtual expert if this advice conflicts with the patient's own previous experience and attitudes in general, eg, that they are opposed to the use of steroids. The lesson learned is that no matter how intelligent and how well supported by acknowledged experts, a computer system cannot replace real face-to-face contact between doctor and patient. In future versions of LinkMedica and similar systems, we suggest that the very detailed (and complicated) feedback system be replaced by a simple "traffic light" approach. For example: "If your asthma diary says green, all is well. If it says yellow two days in a row or more, or red on a single day, contact your asthma doctor."

### The Benefits of Using an Asthma Diary Are Not Recognized Immediately

From classical behavioral psychology we know that in order to reinforce certain behavior, the latency time between behavior and reward must be short. This phenomenon has great implications for treatment of asthma.

In general, it takes time to achieve asthma control. The effect of inhaled steroids on symptoms and exacerbations resulting from previously inadequate treatment may be delayed weeks or months after start of treatment. Furthermore, relapse after cessation of treatment may also be delayed. Since the reward (improved health) is delayed, it can be difficult for the patient to understand why taking regular medication is important. This latency problem is probably one of the reasons that inhaled steroids are being used much less than recommended [19] and also a main reason why patients tend to stop using LinkMedica after a short time.

As with medical treatment, self-management using an electronic asthma diary may take some time to prove its value to the patient. The immediate advantage of measuring peak flow, turning on the computer, connecting to the Internet, logging on LinkMedica, and entering diary values is simply not big enough for the patient to continue doing this for longer periods. Even if a patient has experienced improved asthma control from using LinkMedica, the advantage of continuing use may not (from the patient's viewpoint) justify the inconvenience.

In principle, there are two ways to solve this problem: by improving the accessibility of the diary, which could be achieved by, for example, improved Internet connections, mobile phones, or wireless devices (peak flow meters, electronic dispensing devices, etc.); or by increasing the immediate advantage of diary entry. There may be several ways to achieve the latter. Further studies need to be carried out to reveal whether it is the technology or the lack of immediate advantages that creates obstacles. User studies and explorative design methods [20] can help to clarify the users' needs.

As an example, teenagers would probably adhere more to the diary if they were permitted a small number of free SMS messages for their mobile phones after each diary entry, while others would be encouraged by reimbursement of a portion of their medicine costs linked to how often they entered their diary values. The decrease in unscheduled doctor visits, days off work, etc. resulting from the improved health of users would probably offset the cost of such reimbursement programs. Rewards may also be "virtual": small games where points earned from filling in the diary give access to new game levels might encourage children (and some adults) to adhere to the diary. General information on the user's health-eg, weekly or monthly messages with overall information about the user's asthma, whether it has improved or worsened, and what to do about it-might also prove useful.

### What Can Be Done to Support the Use of LinkMedica and Similar Web Sites?

Given 3 years of experience developing and marketing LinkMedica together with the results of this study, we are able to suggest improvements that would make new users more likely to hold onto their Web-based asthma diaries for longer periods:

- Split LinkMedica into two sites, one for the diary and one for information, discussion groups and ask-the-expert sections.
- The diary should be developed after the "lean-mean-machine" principle, completely free of distracting elements like news, flashy graphics, opinion polls, etc. Fields for login and diary values should preferably be available on the front page, allowing for one-click access and diary entry.
- Redesign the feedback messages to merely *inform* about asthma status, rather than give concrete *advice* regarding dosage etc.
- Remove peak flow from the diary. The value of peak flow monitoring in asthma care is debatable [21], and many users find peak flow measurements cumbersome, which hinders the use of the diary. Alternatively, recording peak flow should be optional.
- Explore other methods for data entry and feedback than conventional Web forms. A combination of daily data entry via mobile phone and occasional Web access for diary overview and graphing facilities could prove valuable.
- Consider how to "reward" users immediately after diary entry. This is essential. Users of the diary must have some sort of immediate reward after dairy entry in order to continue using it for longer periods.

### Conclusion

In general, LinkMedica is regarded as a very reliable and advantageous system by both patients and doctors. However, only a few users are using LinkMedica as intended, and most users, patients as well as doctors, stop using the diary after a short time. There are several reasons for this, the main reason being that the Internet in general and LinkMedica in particular are still not integrated into people's everyday lives. Consequently, if LinkMedica is to become more popular, it needs to be adapted to the conditions of the users, so it becomes a natural and integrated part of their everyday lives.

## Abbreviations

AMC: Asthma Management Centre
GP: General Practitioner
ISDN: Integrated Services Digital Network
KC: Knowledge Centre
SMS: Short Messaging System
GPRS: General Packet Radio Service
